# *Staphylococcus aureus* biofilms decrease osteoblast viability, inhibits osteogenic differentiation, and increases bone resorption *in vitro*

**DOI:** 10.1186/1471-2474-14-187

**Published:** 2013-06-14

**Authors:** Carlos J Sanchez, Catherine L Ward, Desiree R Romano, Brady J Hurtgen, Sharanda K Hardy, Ronald L Woodbury, Alex V Trevino, Christopher R Rathbone, Joseph C Wenke

**Affiliations:** 1 Department of Extremity Trauma and Regenerative Medicine, United States Army Institute of Surgical Research, Ft. Sam Houston, San Antonio, TX, USA

**Keywords:** Biofilm, Osteoblast, Osteogenic differentiation, *Staphylococcus aureus*, Receptor activator of NF-kB ligand, Osteoprotegrin

## Abstract

**Background:**

Osteomyelitis is a severe and often debilitating disease characterized by inflammatory destruction of bone. Despite treatment, chronic infection often develops which is associated with increased rates of treatment failure, delayed osseous-union, and extremity amputation. Within affected bone, bacteria exist as biofilms, however the impact of biofilms on osteoblasts during disease are unknown. Herein, we evaluated the effect of *S*. *aureus* biofilms on osteoblast viability, osteogenic potential, and the expression of the pro-osteoclast factor, receptor activator of NF-kB ligand (RANK-L).

**Methods:**

Osteoblasts were exposed to biofilm conditioned media (BCM) from clinical wound isolates of *Staphylococcus aureus* under normal growth and osteogenic conditions to assess cellular viability and osteoblast differentiation, respectively. Cell viability was evaluated using a live/dead assay and by quantifying total cellular DNA at days 0, 1, 3, 5, and 7. Apoptosis following treatment with BCM was measured by flow-cytometry using the annexin V-FITC/PI apoptosis kit. Osteogenic differentiation was assessed by measuring alkaline phosphatase activity and intracellular accumulation of calcium and osteocalcin for up to 21 days following exposure to BCM. Expression of genes involved in osteogenic differentiation and osteoclast regulation, were also evaluated by quantitative real-time PCR.

**Results:**

BCM from clinical strains of *S*. *aureus* reduced osteoblast viability which was accompanied by an increase in apoptosis. Osteogenic differentiation was significantly inhibited following treatment with BCM as indicated by decreased alkaline phosphatase activity, decreased intracellular accumulation of calcium and inorganic phosphate, as well as reduced expression of transcription factors and genes involved in bone mineralization in viable cells. Importantly, exposure of osteoblasts to BCM resulted in up-regulated expression of RANK-L and increase in the RANK-L/OPG ratio compared to the untreated controls.

**Conclusions:**

Together these studies suggest that soluble factors produced by *S*. *aureus* biofilms may contribute to bone loss during chronic osteomyelitis simultaneously by: (1) reducing osteoblast viability and osteogenic potential thereby limiting new bone growth and (2) promoting bone resorption through increased expression of RANK-L by osteoblasts. To our knowledge these are the first studies to demonstrate the impact of staphylococcal biofilms on osteoblast function, and provide an enhanced understanding of the pathogenic role of staphylococcal biofilms during osteomyelitis.

## Background

Osteomyelitis is a debilitating disease, characterized by the inflammatory destruction of bone and surrounding tissues. Disease is most commonly preceded by hematogenous spread of microorganisms to the bone from either a contiguous infection or directly following trauma. *Staphylococcus aureus* is the microorganism most commonly associated with hematogenous and post-traumatic osteomyelitis, accounting for more than half of all cases [1]. Despite treatment and surgical intervention up to 30% of osteomyelitis cases progress into a chronic infection [2]. Chronic osteomyelitis is associated with high rates of antimicrobial treatment failure, increased rates of non-osseous union and extremity amputation [1,3]. Over the past decade clinicians have adopted the ‘biofilm theory’ to explain the chronicity of bone infections, recalcitrance to conventional antimicrobial treatment, and incidence of infectious relapse [4,5].

Biofilms are surface attached communities consisting of mono- or polymicrobial species that are surrounded by a self produced extracellular polymeric matrix [6,7]. In general, biofilms represent a protected mode of growth enabling the organisms to persist within immunocompetent hosts, and are implicated as a significant pathogenic event in the development of a number of chronic human infections, including osteomyelitis [8,9]. In support of this, studies have demonstrated the presence of staphylococcal biofilms within infected bone of patients with chronic osteomyelitis [9-11], that clinical osteomyelitis isolates of *S*. *aureus* are capable of forming biofilms *in vitro*[12-14], furthermore that staphylococcal biofilms are a significant factor contributing to non-union [15,16]. These studies indicate that staphylococcal biofilms play a critical, yet not fully understood role in the development of chronic osteomyelitis and associated infectious complications.

Osteoblasts function as the major director of net bone formation or resorption during the normal physiological turnover of bone and following infection. Osteoblasts facilitate bone formation directly, through the deposition and calcification of bone matrix promoting new bone growth, and promote bone resorption indirectly by regulating the activity of osteoclasts by the production of two cytokines, the receptor activator of NF-kB ligand (RANK-L) and osteoprotegrin (OPG), which promote and block osteoclast activity respectively [17-19]. During infection, shifts in the ratio of these two cytokines can contribute to overall bone loss, which is typical of chronic orthopaedic infections. This is largely due to the combined effects of microorganisms to elicit a local host inflammatory response and impact osteoblast function [20,21]. The current understandings of the molecular mechanisms that contribute to bone loss during osteomyelitis are based on evaluating interactions between planktonic bacteria and osteoblasts *in vitro*[20,22-24]. While these studies have provided insight into the bacterial mechanisms contributing to bone loss, they fail to address the role of the biofilm phenotype during disease, which is likely to be more representative of the bacterial mode of growth *in vivo*[4,8,9]. Because osteoblasts play a critical role for bone homeostasis and healing, a logical speculation is that biofilms interacting with these tissues during disease may exert their negative effects on this cell type.

In this study, we evaluated the effect of *S*. *aureus* biofilms on viability, osteogenic potential, and the production of RANK-L by human osteoblasts in response to biofilms. Herein, we characterize the extracellular proteome of released factors produced by *S*. *aureus* biofilms and demonstrate that biofilm-derived factors negatively impact cell viability and osteoblast differentiation. Furthermore, exposure of osteoblasts to biofilm-conditioned media also resulted in increased expression of RANK-L and the RANK-L/OPG ratio, indicating that biofilms simultaneously promote bone resorption. These studies are the first to show the effect of biofilms on osteoblast function and to demonstrate mechanisms through which biofilms may contribute to bone loss during chronic osteomyelitis.

## Methods

### Bacterial strains and growth conditions

*S*. *aureus* SAMMC-700 is a methicillin resistant clinical wound isolate of the USA300 clonal group recovered from a patient as a part of treatment and not related to research from the San Antonio Military Medical Center (SAMMC, Ft. Sam Houston, TX). UAMS-1 (ATCC strain 49230) is a methicillin-susceptible *S*. *aureus* strain of the USA200 clonal group, and a well characterized osteomyelitis isolate [25,26]. Both clinical strains were included in this study to thoroughly examine the impact of staphylococcal biofilms on osteoblast function. Bacteria were cultured in Tryptic Soy Broth (TSB) or agar plates overnight at 37°C. For planktonic growth, individual colonies from overnight plate cultures of bacteria were used to inoculate TSB broth and were grown with agitation at 37°C.

### Biofilm formation and preparation of biofilm-conditioned media (BCM)

Biofilm formation and generation of conditioned media were performed as previously described [27,28]. Briefly, 500 μL of a 1:100 dilution of overnight bacterial culture (~10^7^ CFU/mL) was added to transwell culture inserts (0.4 μm pore size, Corning Inc, Corning, NY) and grown under static conditions in TSB broth for 48 hr at 37°C. Inserts were washed for 1 hr in 1× phosphate buffered saline (PBS), placed into Dulbecco’s Modified Eagle’s Medium (DMEM) (Invitrogen, Carlsbad, CA) and maintained in DMEM at 37°C for an additional 4 days. BCM was collected daily and replaced with fresh media. Pooled BCMs were filter-sterilized using a 0.2 μm syringe filter (EMD Millipore, Billerica, MA), pH adjusted to 7.4, and stored at −80°C until use. Sterility of collected BCM was assessed by spot plating and overnight culture of the plates. Total protein from pooled BCM was quantified using the bicinchonic acid (BCA) assay (Pierce, Rockford, IL) following the manufacturers protocol. Because *S*. *aureus* SAMMC-700 is an uncharacterized clinical wound isolate, further characterization by mass spectroscopy was pursued for this BCM. The BCM of *S*. *aureus* UAMS-1 was included for cellular experiments for a more extensive study.

### Scanning electron microscopy (SEM)

For SEM analysis tissue culture inserts were excised, fixed with 2% (w/v) glutaraldehyde, 2% (w/v) paraformaldehyde (PFA), 0.15 M sodium cacodylate, 0.15% (w/v) alcian blue for 3 hr, rinsed 3× with 0.15 M sodium cacodylate buffer, and incubated in 1% (v/v) osmium tetroxide in sodium cacodylate for 1 hr. Samples were dehydrated with a stepwise gradient of ethanol and then treated with hexamethyldisilizane prior to drying in a desiccator overnight. Samples were sputter coated with gold palladium and viewed with a JEOL-6610 scanning electron microscope (JEOL USA, Inc., Peabody, MA).

### Protein identification by mass spectrometry

Cell lysates were loaded onto a pre-cast 12% SDS-PAGE gel (Bio-Rad, Hercules, CA) in Laemmli sample buffer and run 2 cm into the gel as measured from the bottom of the well. The gel was fixed and stained for 60 min using colloidal coomassie and de-stained overnight in water. Lanes were evenly cut into 6 slices starting at the top of the resolving gel and ending at the dye front (~1 cm). Each slice was further diced into 1 mm × 1 mm cubes. Slices were then digested *in situ* with trypsin according to standard protocols based on the initial work of Mann and co-workers [29]. Briefly, protein bands were excised from the gel and destained twice in 50% acetonitrile (ACN)/40 mM ammonium bicarbonate, pH 7.4, prior to digestion. Gel plugs were then dehydrated in 100% ACN and rehydrated with 5 to 10 μl of 10 ng/μl trypsin (Promega; modified) in 40 mM ammonium bicarbonate/20% ACN and incubated overnight at 30°C. The resulting peptides were extracted in 4 volumes of 0.1% TFA/50% ACN for 1 to 2hr at RT, decanted from the gel slice, dried down in an autosampler tube in the speed vacuum w/o heat, and resuspended in 0.5% TFA. Peptides were analyzed by capillary-HPLC-electrospray tandem mass spectrometry (HPLC-ESI-MS/MS) on a Thermo Fisher LTQ ion trap mass spectrometer coupled to an Eksigent NanoLC micro HPLC by means of a PicoView (New Objective) nanospray interface. Capillary on-line HPLC separation of tryptic peptides was conducted using the following conditions: column, New Objective PicoFrit, 75 μm id, packed to 11 cm with C18 adsorbent, (Vydac 218MSB5); mobile phase A, 0.5% acetic acid/0.005% TFA in water; mobile phase B, 90% ACN/0.5% acetic acid/0.005% TFA in water; gradient, 2% B to 42% B in 30 min; flow rate, 0.4 μl/min. A data-dependent acquisition protocol was employed consisting of one survey scan followed by 7 collision-induced dissociation spectra. The un-interpreted CID spectra were searched against the NCBInr database using Mascot (Matrix Science; 10 processor in-house license). Methionine oxidation was the only variable modification considered. Maximum missed cleavages for trypsin was set at 1, peptide charge at 2+ and 3+, peptide tolerance at +/− 1.5 Da, and MS/MS tolerance at +/− 0.8 Da. Mascot data was then run in Scaffold 3.1 (http://www.proteomesoftware.com) and cross correlation of the Mascot results was carried out by X! tandem against the NCBInr subset database. Proteins with an expectation score of 10^-3^ or lower were considered positive identities. Proteins were identified with 3–15 matched peptides and a minimum of 95% sequence coverage.

### Culture of human osteoblasts and osteogenic differentiation

Human osteoblasts (PromoCell, Heidelberg, Germany) were maintained in DMEM supplemented with 10% fetal bovine serum (FBS), penicillin 10U mL^-1^ and streptomycin 10 μg mL^-1^ at 37°C in 5% CO_2_. For osteogenic differentiation of osteoblasts cells were seeded into 24-well plates or 10 cm culture dishes and when cells reached 80% confluence they were cultured in media supplemented with ascorbic acid (50 μM), β-glycerolphosphate (20 mM) and dexamethasone (1.5 μM) (Sigma, St. Louis, MO) for up to 21 days in the presence or absence of BCM.

### Cell viability and proliferation

Cell viability and proliferation of osteoblasts was assessed using a LIVE/DEAD^©^ assay kit and by quantifying total cellular DNA using the CyQUANT® Cell Proliferation Assay (Molecular Probes, Grand Island, NY), respectively, following exposure to BCM up to 21 days in 48-well plates as recommended by the manufacturer.

### Measurement of apoptosis in osteoblasts

Apoptosis in osteoblasts was evaluated after 24 hr treatment with BCM using the Annexin V-FITC apoptosis detection kit (BD, Franklin Lakes, NJ) by fluorescence-activated cell sorting (FACS) using a FACSCalibur flow cytometer (BD, Franklin Lakes, NJ) as previously described [30]. Data was analyzed using FlowJo software (Tree Star, Inc., Ashland, OR). As a positive control for apoptosis, 4 μM staurosporine (Sigma, St. Louis, MO) was used [31].

### Alkaline phosphatase (ALP) measurement

Quantification of ALP activity in osteoblasts was carried out using the SensoLyte® pNPP Alkaline Phosphatase Assay (AnaSpec, Fremont, CA) according the manufacturer’s protocol. Briefly, cells were lysed using Triton X-100, and alkaline phosphatase activity was detected within supernatants by measuring absorbance of the dephosphorylated chromogenic substrate, p-Nitrophenyl phosphate, at 405 nm. Concentrations of ALP within the supernatants were determined using a standard curve generated with calf intestinal ALP.

### Osteocalcin staining

Osteoblasts were grown and differentiated for 14 and 21 days in 24-well plates in the presence or absence BCM as above. Cells were then fixed with 4% PFA, permeabilized and blocked with 1% BSA + 0.1% Triton X-100 in 1× PBS for 30 min. Mouse anti-human antibody for osteocalcin (R&D systems, Minneapolis, MN) was diluted 1:200 and added to cells for 1 hr. Phycoerythrin (PE) labeled secondary goat anti-mouse antibody (Abcam, Cambridge, MA) was added at a final concentration of 1:10,000 and cells were visualized with an Olympus IX71 inverted fluorescence microscope (Olympus Inc, Center Valley, PA).

### Alizarin red S staining

Intracellular calcium deposition in osteoblasts following treatment with BCM under differentiating conditions was assessed by Alizarin Red staining. Briefly, osteoblasts were seeded into 24-well plates and grown under osteogenic conditions as described above in the presence or absence of BCM. At 7, 14, and 21 days cells were fixed with 4% PFA, washed with sterile 1× PBS, stained with 2% (w/v) Alizarin Red S (Sigma, St. Louis, MI) and visualized by light microscopy.

### RNA extraction and real-time PCR

RNA was extracted from cells treated with BCM in 10 cm cell culture dishes, processed with the QIAshredder, and purified using the RNAeasy Mini Kit (Qiagen, Valencia, CA) as per manufacturer instructions. First strand synthesis was achieved with SuperScript III first-strand synthesis supermix with oligo-dT primers (Invitrogen, Carlsbad, CA) for each RNA sample following recommended protocols using a PTC-100 Thermal Cycler (GMI Inc, Ramsey, MN). For genes of interest (Table 1), quantitative real-time polymerase chain reaction (qRT-PCR) was performed using a Bio-Rad C1000 system and analyzed using iQ5 software (BioRad, Hercules, CA). The primers sets used in this study were based on optimized and validated primers from PrimerBank^©^ (Table 1). Amplification reactions were performed using qPCR iQSYBR Green Super Mix (BioRad, Hercules, CA) with the following conditions: 10 min at 95°C, followed by 40 cycles at 95°C for 10s, 60°C for 30 s, 72°C for 30 s. Three independent biological experiments with three technical replicates were performed for each reaction. Transcript levels were normalized to the internal control, Glyceraldehyde-3-phosphate dehydrogenase (GAPDH), mRNA and changes in relative expression were calculated using 2^–ΔCt^ method [32].

**Table 1:** **Primers used in this study**^***a***^

**Gene**	**Accession number**	**Primer sequence**
Activating transcription factor 4	ATF	Sense CCCTTCACCTTCTTACAACCTC
	(NM_182810)	Antisense TGCCCAGCTCTAAACTAAAGGA
Runt-related transcription factor 2	RUNX2	Sense TGGTTACTGTCATGGCGGGTA
	(NM_001015051)	Antisense TCTCAGATCGTTGAACCTTGCTA
Alkaline phosphatase	ALP	Sense AACATCAGGGACATTGACGTG
	(NM_001127501)	Antisense GTATCTCGGTTTGAAGCTCTTCC
Osteocalcin	BGLAP	Sense CACTCCTCGCCCTATTGGC
	(NM_000711)	Antisense GCCTGGGTCTCTTCACTACCT
Osteonectin	SPARC	Sense CCCATTGGCGAGTTTGAGAAG
	(NM_003118)	Antisense AGGAAGAGTCGAAGGTCTTGTT
Receptor Activator of NF-KB Ligand	RANK-L	Sense GTCTGCAGCGTCGCCCTGTT
	(NM_003701)	Antisense ACCATGAGCCATCCACCATCGC
Osteoprotegerin	OPG	Sense CGCCTCCAAGCCCCTGAGGT
	(NM_002546)	Antisense CAAGGGGCGCACACGGTCTT
Glyceraldehyde-3-phosphate	GAPDH	Sense CAGCCTCCCGCTTCGCTCTC
	(NM_002046)	Antisense CCAGGCGCCCAATACGACCA

^***a***^Primers used in this study were obtained from Primer Bank “PCR Primers for gene Expression Detection and Quantification”. http://pga.mgh.harvard.edu/primerbank/ (The Center for Computational and Integrative Biology). All selections were made relative to comparable annealing temperatures, as well as products close to 100bp in length to maintain consistent PCR conditions.

### Statistical analysis

Statistical analyses were performed using One-way ANOVA with appropriate post-hoc tests for comparisons between groups using sigmaplot version 12.0. *P*-values of <0.05 were considered to be statistically significant.

## Results

### Characterization of biofilm-derived factors produced by clinical isolates of *S*. *aureus*

**Figure 1:**
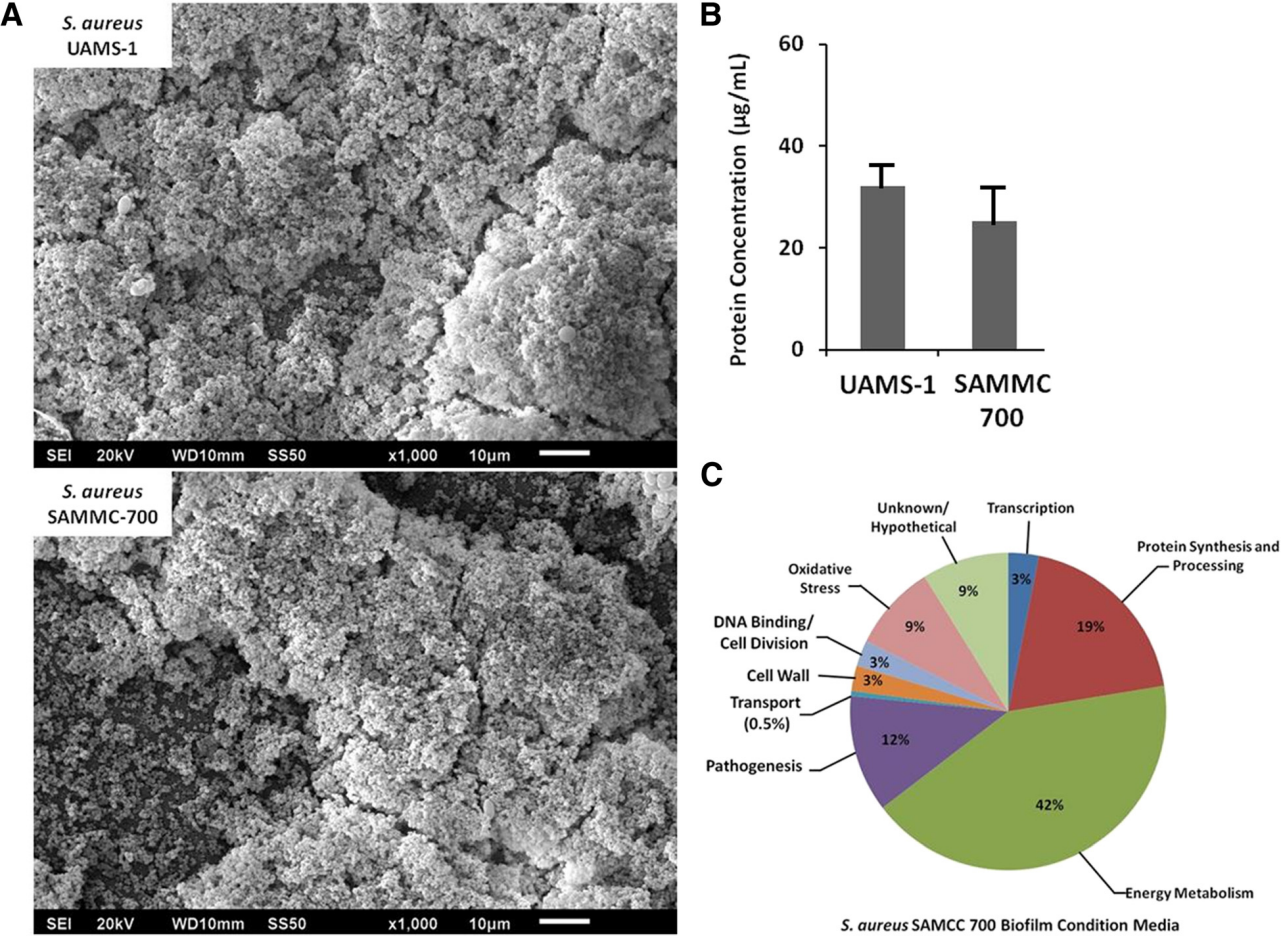
**Soluble factors produced by *Staphylococcus aureus *biofilms. A**) Representative SEM images of *S*. *aureus* strain UAMS1 (top panel) and SAMMC −700 (bottom panel) after 48 hr growth (scale bars =10 μm). **B**) Total protein (μg/mL) within the BCMs of *S*. *aureus* clinical strains as determined by the BCA assay. **C**) Proteomic analysis of BCM from *S*. *aureus* strain SAMMC-700 by mass spectroscopy. Proteins identified within the BCM were separated into functional categories, and represented as a percentage of the total proteins identified.

After 48 hours of growth under static conditions, both *S*. *aureus* strain UAMS-1 and SAMMC-700 formed mature biofilms on the transwell surface. As shown in the representative SEM images, mature biofilms of both strains were characterized by large heterogeneous structures composed of accumulations of aggregated bacteria, with few visible planktonic bacteria, confirming that the majority of cells using this model were in a biofilm state (Figure 1A). BCM on average contained 32 ± 5 and 25 ± 6 μg/mL of extracellular protein for UAMS-1 and SAMMC-700 respectively (Figure 1B). Given the similarity in protein concentrations between the BCM produced by both strains; moreover that UAMS-1 is a well characterized isolate, we choose to characterize the BCM of the wound isolate SAMMC-700 by mass spectrometry to identify those soluble factors present. Proteomic analysis of the biofilm conditioned media (BCM) from *S*. *aureus* SAMMC-700, demonstrated the presence of a heterogeneous mixture of proteins encompassing all aspects of bacterial physiology, including transcription, translation, energy metabolism, pathogenesis, and proteins of unknown function (Figure 1C; Additional file 1). The predominate class of extracellular proteins present within the BCM were primarily those involved in energy metabolism (42%), including carbohydrate, lipid, and nucleotide metabolism, as well as those involved in protein synthesis and processing (19%). Interestingly, although virulence factors were detected within the BCM of *S*. *aureus* SAMMC-700, this group represented a small percentage of the total extracellular proteome (12%; Additional file 1).

### Biofilm-derived factors reduce osteoblast viability by activating apoptosis

**Figure 2:**
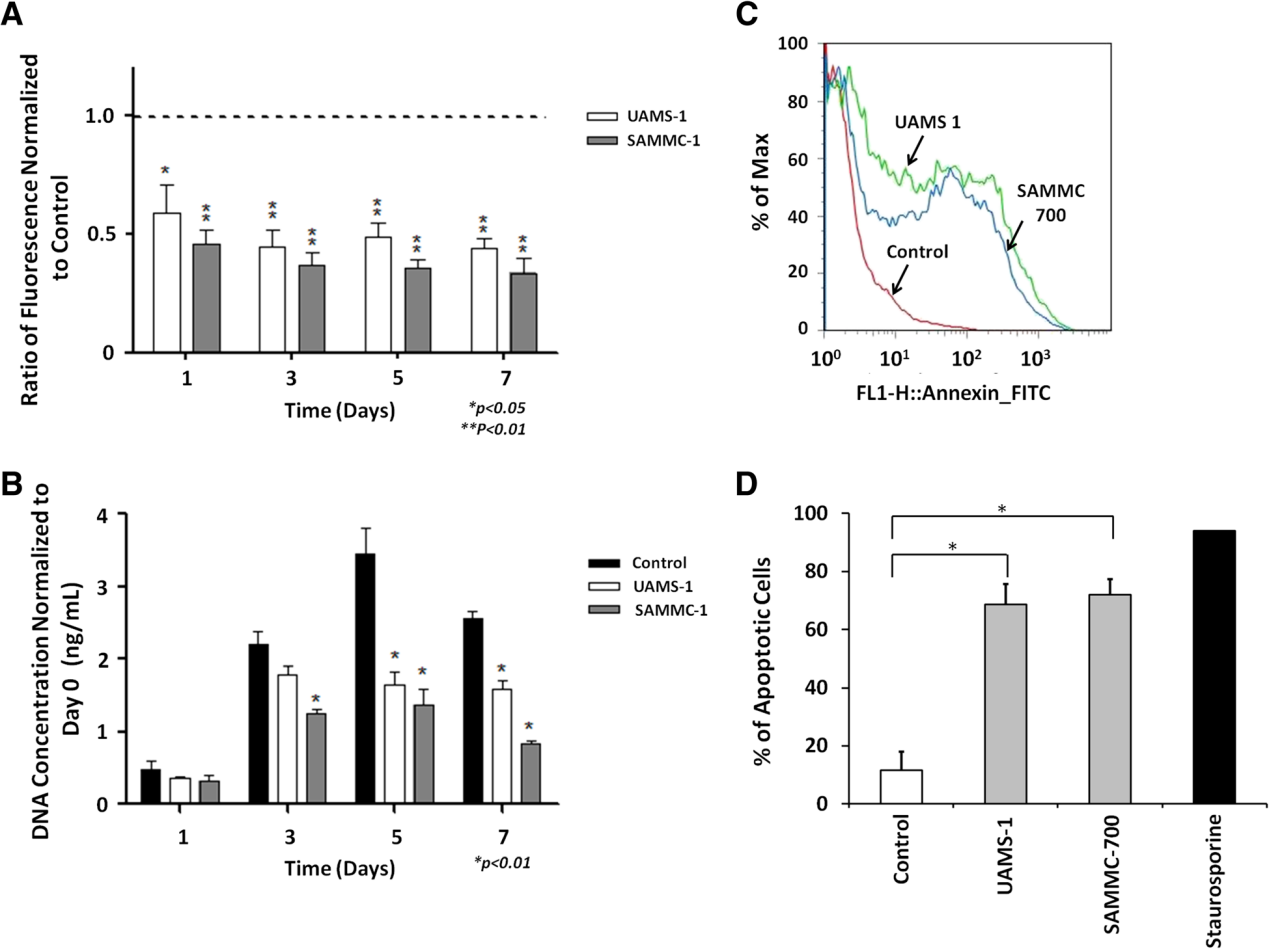
**Biofilm factors reduce viability and activate apoptosis in human osteoblasts.A**) Viability in osteoblast expressed as ratio of fluorescence (495ex/515em) normalized to non-treated control group **B**) Total DNA recovered from osteoblasts following treatment with BCM, normalized to day 0 controls. Bars represent experimental averages of three independent experiments ± standard deviation. **C**) Representative flow-cytometry histograms measuring apoptosis in osteoblasts exposed to BCM for 24 hr by Annexin V staining and **D**) corresponding percentages of Annexin V positive cells from two independent experiments. Statistical analysis was performed using a One-Way ANOVA with a Bonferroni test to determine statistical differences between groups; *p<0.01, ** p<0.001 versus controls.

BCMs from *S*. *aureus* UAMS-1 and SAMMC-700 had a dose-dependent effect on the viability of human osteoblasts, with concentrations ≥ 50% having a significant, detrimental effect on cell viability within 24 hr. In contrast, at 25% minimal effects on osteoblast viability were observed albeit increasing over time with maximal effect at 21 days, whereas concentrations 25% had little to no effect at 24 hr (Additional files 2, 3). Based on the viability studies and to permit the adequate time required to evaluate osteogenic differentiation, we chose to use the 50% BCM for short term (≤ 7 days) studies, including viability and apoptosis, and 25% BCM for extended experiments (≥ 7 days), those related to evaluating osteogenic differentiation. Exposure of osteoblasts to the BCM from *S*. *aureus* SAMMC-700 significantly reduced cell viability and proliferation as determined by measuring the amount of viable cells and quantification of cellular DNA, respectively (Figure 2A-B). Evaluation of osteoblasts, following treatment with BCM for 24 hr by Annexin V staining, demonstrated that BCM of *S*. *aureus* was capable of inducing apoptosis (Figure 2C-D). These results suggest that the decreases in cell number may have been due, at least in part, to an increase in apoptosis. Importantly, the effects of BCM on osteoblasts were not strain independent, as similar effects on viability and activation of apoptosis were observed when using the BCM of *S*. *aureus* strain UAMS-1 (Figure 2A-D).

### Staphylococcal biofilm factors inhibit osteogenic differentiation *in vitro*

**Figure 3:**
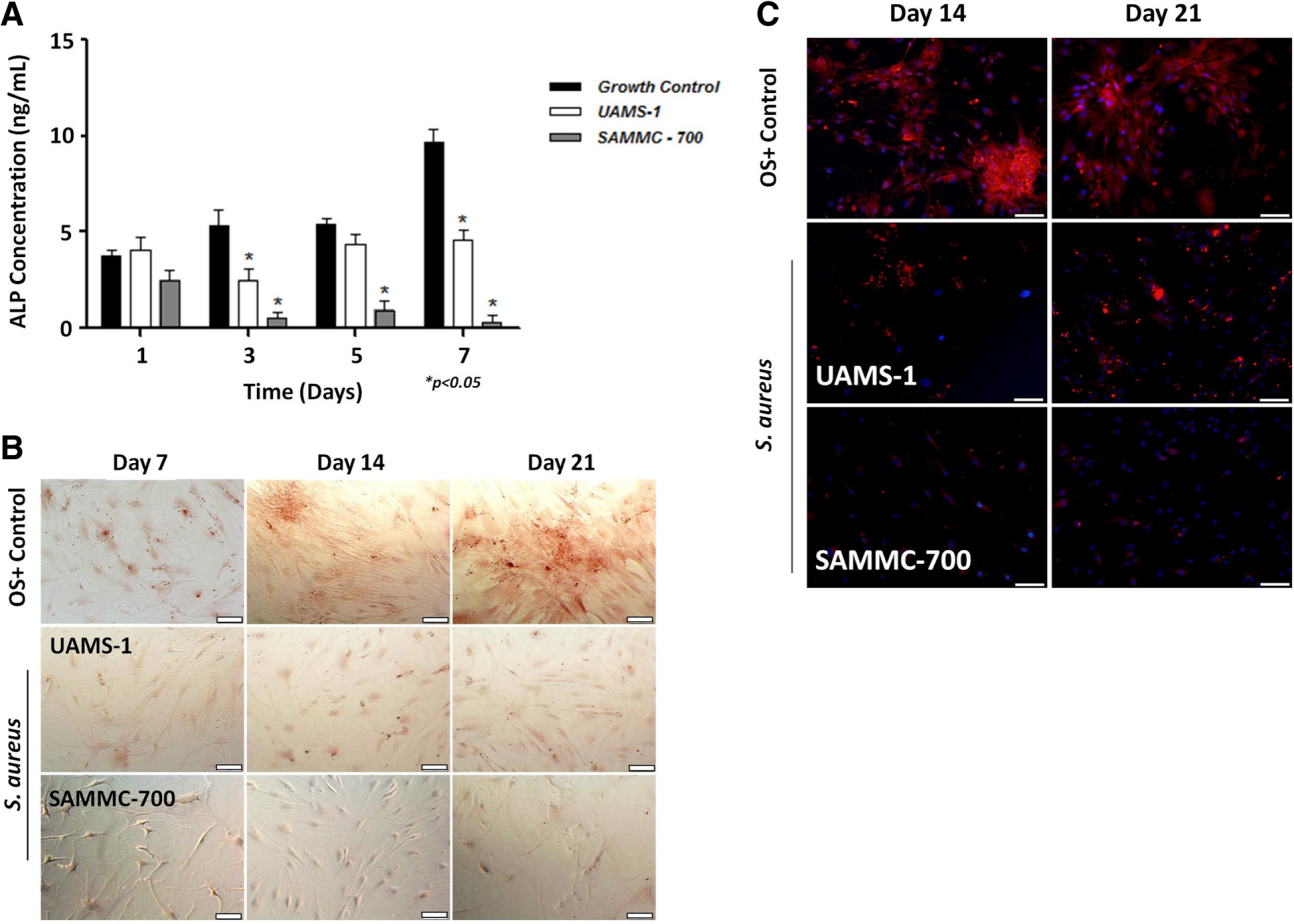
**Staphylococcal biofilm factors inhibit osteogenic differentiation in human osteoblasts. A**) ALP concentration in osteoblasts exposed to BCM. Bars represent the averages ± std dev from three independent experiments. **B**) Calcium deposition in osteoblasts and **C**) osteocalcin in osteoblasts treated with BCM revealed by Alizarin Red S staining and immunofluoresence using specific antibody, respectively. Images were taken at 10× magnification (bars represent 100 μm). Statistical analysis was performed using a One-Way ANOVA analysis using a Bonferroni test to determine statistical differences between groups.* p< 0.01 versus control group.

To evaluate the effect of soluble factors produced by biofilms on osteogenic differentiation, a critical function of this cell type, osteoblasts were cultured under osteogenic conditions in the presence or absence of BCM for up to 21 days. The addition of BCM from the two clinical strains of *S*. *aureus* dramatically reduced ALP activity (Figure 3A) as well as the intercellular accumulation of calcium and osteocalcin (Figure 3B, C) in viable cells when compared to the osteogenic control group. Consistent with these results, gene expression analysis of the transcriptional regulators involved in osteogenic differentiation, including activating transcription factor 4 (*Atf4*), runt-related transcription factor 2 (*Runx2*), as well as those genes involved in matrix mineralization, alkaline phosphatase (*Alp*), osteocalcin (*Bglap*) and osteonectin (*Sparc*) by qRT-PCR were also observed to be significantly decreased at days 7 and 14 following treatment with the BCM in viable cells (Table 2).

**Table 2:** Relative expression of genes involved in osteogenic differentiation

	**Day 7**		**Day 14**	
	**Relative expression**	**Fold difference**^**a**^	**Relative expression**	**Fold difference**^**a**^
**Transcription factors**				
***runx2***				
No Treatment (+ osteogenic media)*	1.12 ± 0.10	5.333	2.55 ± 0.23	3.643
UAMS-1	0.12 ± 0.18	0.590	0.93 ± 0.22	1.329
SAMMC-700	0.08 ± 0.25	0.381	0.13 ± 0.80	0.186
***atf4***				
No Treatment (+ osteogenic media)*	35.4 ± 10.0	2.476	72.1 ± 0.30	3.433
UAMS-1	16.1 ± 0.15	1.126	26.1 ± 1.00	1.243
SAMMC-700	13.5 ± 0.16	0.944	19.3 ± 0.21	0.919
**Genes involved in mineralization**				
***alp***				
No Treatment (+ osteogenic media)*	5.73 ± 0.14	6.374	9.01 ± 0.04	15.615
UAMS-1	0.17 ± 0.01	0.187	1.12 ± 0.04	2.166
SAMMC-700	0.27 ± 0.01	0.306	0.21 ± 0.10	0.406
***bglap***				
No Treatment (+ osteogenic media)*	0.77 ± 0.04	6.814	6.0 ± 0.33	40.816
UAMS-1	0.001 ± 1.00	0.009	0.10 ± 0.33	0.680
SAMMC-700	0.001 ± 0.51	0.009	0.01 ± 0.34	0.068
***sparc***				
No Treatment (+ osteogenic media)*	174 ± 37.8	2.568	124 ± 0.21	1.879
UAMS-1	44.5 ± 22.7	0.656	48.7 ± 0.24	0.738
SAMMC-700	35.7 ± 24.2	0.526	84.9 ± 0.25	1.286

^a^fold difference is the difference in relative gene expression compared to osteoblasts grown under. non-osteogenic conditions (− osteogenic media).

*Indicates a positive osteogenic differentiation control; osteogenic media no BCM treatment.

### Biofilm conditioned medias promote bone resorption by up regulating RANK-L expression and increasing the RANK-L/OPG ratio

**Figure 4:**
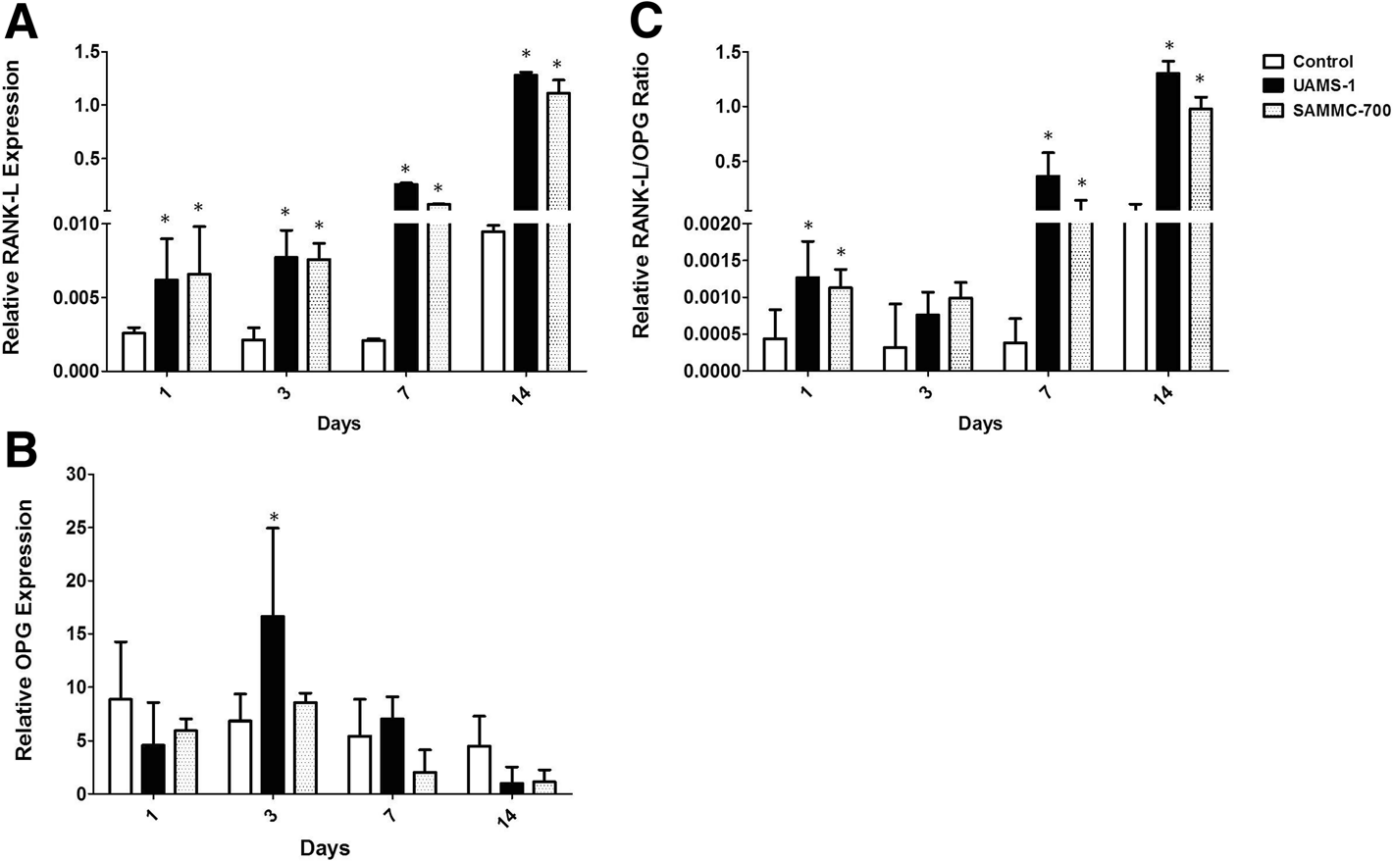
**Biofilm-derived factors increase the expression of RANK-L and the RANK-L/OPG ratio in human osteoblasts.** Relative gene expression of **A**) RANK-L, **B**) OPG, and **C**) the RANK-L/OPG ratio in osteoblasts exposed to BCM for 1, 3,7 and 14 days. Gene expression levels were measured by qRT-PCR, normalized to the internal control GAPDH, and presented as relative expression using the 2^-ΔCT^ method. Bars represent averages ± standard deviation from three independent experiments. Statistical analysis was performed using a One-Way ANOVA analysis using a Bonferroni test to determine statistical differences between groups at each timepoint; * p< 0.01 versus control group.

To assess the effect of staphylococcal biofilm factors on RANK-L and OPG expression in osteoblasts, we evaluated relative gene expression by qRT-PCR analysis following exposure to BCM. At 1, 3, 7 and 14 days relative levels of RANK-L expression in osteoblasts were significantly increased in comparison to the control group following exposure to the BCM of both *S*. *aureus* strains (Figure 4A). Relative expression of OPG was increased at day 3 but not at any of the other days evaluated for UAMS-1(Figure 4B). No significant increases of OPG were observed in osteoblasts treated with BCM from *S*. *aureus* strain SAMMC-700. Importantly, compared to the control group BCM from both strains of *S*. *aureus* significantly increased the RANK-L/ OPG ratio in osteoblasts at days 1, 7 and 14 (Figure 4C). BCMs of *S*. *aureus* strains SAMMC-700 and UAMS-1 increased the RANK-L/OPG ratio 3.7- and 4.6-fold at day 1, an average of 1.5-fold at day 3, and averages of >40 fold at days 7 and 14.

## Discussion

Bone regeneration following normal physiological turnover and pathological events requires coordinated responses directed by osteoblasts to promote adequate resorption and new bone growth. The depletion of osteoblasts, inhibition of osteogenic potential, or dysregulation of bone resorption by microorganinisms during infection, such as *S*. *aureus*, can have detrimental effects on osseous regeneration. The current understanding of pathological mechanisms of bone loss during bacterial disease is from studies evaluating interactions between planktonic bacteria and osteoblasts [20,24,33-35]. Although these studies have provided insight into some of the pathological mechanisms contributing to bone loss they do not address the role of the biofilm during disease. This is a particularly important lapse, given that staphylococcal biofilms are considered to be the predominate mode of growth in affected bone *in vivo*, and have a central, yet not fully understood role, in the development of chronic osteomyelitis [4,8,15,16].

Microbial biofilms represent a significant barrier to wound healing [36-38]. The ability of biofilms to delay wound healing is well characterized and has been shown, in part, to be a direct effect of soluble bacterial products released from the biofilm on host cells [27,39,40]. For example, supernatants from *S*. *aureus* biofilms have been shown to reduce cellular migration and induce apoptosis in fibroblasts and keratinocytes [27,39]. Likewise, supernatants from *Pseudomonas aeruginosa* and *Enterococcus spp*. have also been shown to inhibit keratinocyte migration and other wound healing functions [41]. Osteoblasts are the primary cell type involved in facilitating new bone growth and the depletion of this cell type during chronic disease can have a tremendous impact on healing outcome. The exposure of osteoblasts to BCM of *S*. *aureus* was observed to significantly reduce viability, which was in part, due to activation of apoptosis. Previous studies have shown that when co-cultured with planktonic *S*. *aureus*, osteoblasts undergo apoptosis following internalization of the bacterium, due to activation of TNF-related apoptosis inducing ligand (TRAIL) [35,42], and more recently through a family of nucleotide binding domain leucine-rich repeat region (NLRs) proteins, including NLRP-3 [43]. In contrast to studies with planktonic bacteria, our results indicate that *S*. *aureus* biofilms activated apoptosis in osteoblasts through a paracrine effect. As TRAIL and NLRP-3 activation require active infection of the host cell, these findings indicate that a separate mechanism may be involved in activating apoptosis in osteoblasts following exposure to soluble factors produced by biofilms. Future studies are necessary to characterize the mechanisms through which apoptosis is activated in osteoblasts following exposure to BCM.

Analysis of the extracellular proteome of the BCM of *S*. *aureus* strain SAMMC-700 demonstrated the presence of a heterogeneous mixture of proteins. This included proteins primarily involved in energy metabolism, protein synthesis and to a lesser extent those involved in virulence, oxidative stress, and transcription (Additional file 1). The predominance of cytosolic proteins within the BCM is likely the result of the bacterial autolysis that occurs naturally during the maturation of the biofilm [44,45]. Although relationships between bacterial metabolism and virulence have been reported, soluble virulence factors, including alpha (α)-hemolysin, gamma (γ) –hemolysin and staphopain B, detected within the BCM may represent those factors likely contributing to cellular apoptosis. *Staphylococcus aureus* produces a number of different components contributing to cellular toxicity, including exoenzymes and exotoxins. Exotoxins produced by *S*. *aureus* include a family of four membrane-damaging hemolysins (alpha-, beta-, gamma-, and delta-hemolysin) of which α-hemolysin (*Hla*) plays an important role in the pathogenesis [46]. Alpha-toxin is a pore-forming hemolytic toxin that causes membrane damage and is capable of inducing apoptosis in a wide range of mammalian cells including, keratinocytes [47], fibroblasts [48], and monocytes [49]. Likewise, the staphylococcal cysteine proteinase, staphopain B (SspB) critically impairs antibacterial functions and induces cell death in neutrophils and monocytes [50,51]. The impact of staphylococcal hemolysin or staphopain on osteoblast has not been previously described, and may represent an uncharacterized role for these virulence factors during osteomyelitis. Although the activation of apoptosis in osteoblasts following exposure to BCM is strongly suggestive of a role for cytolysins and/or the staphopain this does not exclude the possibility that other metabolites and/or proteins released by the biofilms may contribute to the effects on osteoblasts. The identification of those factors that mediate these effects on osteoblasts are currently underway.

Osteoblasts facilitate new bone growth by depositing and facilitating the calcification of bone matrix. During osteoblast differentiation, several markers are expressed including alkaline phosphatase, which are important for bone matrix deposition and mineralization, as well as regulators of matrix calcification, including osteocalcin and osteonectin. Notably, the differentiation of osteoblasts is highly regulated by the temporal activation of various transcriptional regulators of which RUNX2 is best described [52]. RUNX2 is a transcription factor that controls skeletal development by regulating osteoblast differentiation and expression of many extracellular matrix protein genes during osteogenesis, including alkaline phosphatase (*Alp*) and osteocalcin (*Bglap*) [52,53]. In the presence of BCM osteogenic differentiation was significantly impaired. Based on previous studies indicating the crucial role of the transcriptional regulators, *Cbfa1*/*Runx2*, during osteogenic differentiation our results suggest that BCMs may affect osteoblast function by decreasing the expression of *Runx2* in viable, non-apoptotic cells. Whether other pathways involved in osteogenic differentiation, including WNT signaling among others, are impacted by BCM remain to be determined.

In addition to supporting bone growth, osteoblasts also regulate bone resorption. This is accomplished through indirect control of osteoclast activity through the relative ratio of RANK-L/OPG. In the presence of BCM, RANK-L expression and the RANK-L/OPG ratios were significantly increased compared to control groups as early as day. Increased expression of RANK-L and/or changes in RANK-L/OPG ratio have been shown to be strong predictors of rapid and persistent bone loss in rheumatoid arthritis, osteoporosis, and periodontal disease [22,54]. Consequently, during disease biofilms, in addition to reduced osteogenesis, simultaneously may promote bone loss through activation of osteoclast activity as a result of increased RANK-L production by osteoblasts. The combined effects of biofilms on osteoblasts can have a significant impact on disease progression and the healing outcome in the patient.

## Conclusion

Biofilm formation is a central event in the development of chronic bone infections and has been shown to be a significant contributing factor to non-osseous union in patients with osteomyelitis. The results from this study indicate that soluble biofilm factors from clinical isolates of *S*. *aureus* can affect bone formation and resorption through simultaneous mechanisms including: 1) reducing cellular viability through apoptosis, 2) inhibition of osteogenic differentiation, and 3) increasing RANK-L expression, promoting bone resorption. These studies are the first to demonstrate the impact of biofilms on osteoblasts; moreover to provide insights into the pathogenic mechanisms of biofilms that contribute to infectious complications during chronic bone infections.

## Competing interests

The authors declare that they have no competing interests.

## Authors’ contributions

CJS participated in the study design, experimental studies and data analysis, and wrote the final draft of this manuscript. CLW participated in the study design, experimental studies, data analysis, and in reviewing manuscript. DRR, BJH, SKH, RLW and AVT performed the experimental studies and acquisition of data. CRR and JCW participated in the study design, data analysis, and helped review the manuscript. All authors read and approved the final version of the manuscript.

## Pre-publication history

The pre-publication history for this paper can be accessed here:

http://www.biomedcentral.com/1471-2474/14/187/prepub

## Supplementary Material

Additional file 1: Table S1 Soluble factors identified within BCM of S. aureus SAMMC-700 by mass spectroscopy. Click here for file

Additional file 2 **Dose dependent response of BMC on osteoblast viability.** osteoblasts were exposed to various concentrations (5, 10, 15, 25, 50, 75 and 100%) of BMC for up to 3 days. Viability following exposure was determined by measuring fluorescence (495ex/515em) and expressed as a ratio to the non-treated control group. Click here for file

Additional file 3 **Effect of biofilm conditioned media (BMC) on Osteoblast Viability.** Osteoblast were exposed to BMC at 25% for up to 21 days. Viability following exposure was determined by measuring fluorescence (495ex/515em) and expressed as a ratio to the non-treated control group. Click here for file

## Disclaimer

The views expressed herein are those of the authors and do not reflect the official policy or position of the Department of the Army, Department of Defense, or the US Government. The authors are employees of the US government. This work was prepared as part of their official duties and, as such, there is no copyright to be transferred.
