# Supplementary figures and images for: Staphylococcus aureus biofilms decrease osteoblast viability, inhibits osteogenic differentiation, and increases bone resorption in vitro
Source: BMC Musculoskelet Disord. 2013 Jun 14;14:187. doi: 10.1186/1471-2474-14-187 (PMC3691632; doi:10.1186/1471-2474-14-187)

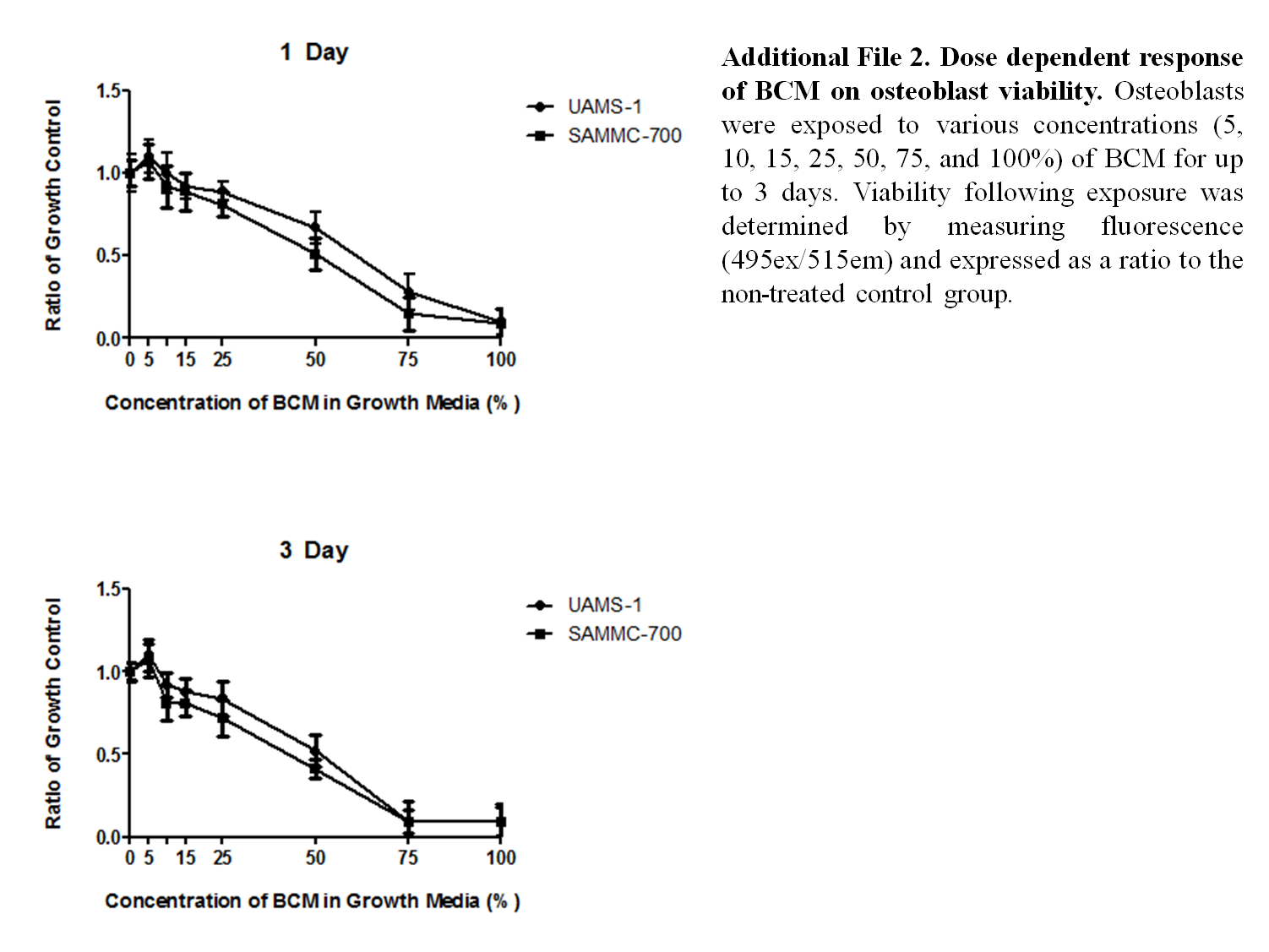

Supplement: Additional file 2 — Dose dependent response of BMC on osteoblast viability. osteoblasts were exposed to various concentrations (5, 10, 15, 25, 50, 75 and 100%) of BMC for up to 3 days. Viability following exposure was determined by measuring fluorescence (495ex/515em) and expressed as a ratio to the non-treated control group. [file 1471-2474-14-187-S2.tiff]

## Slide 1
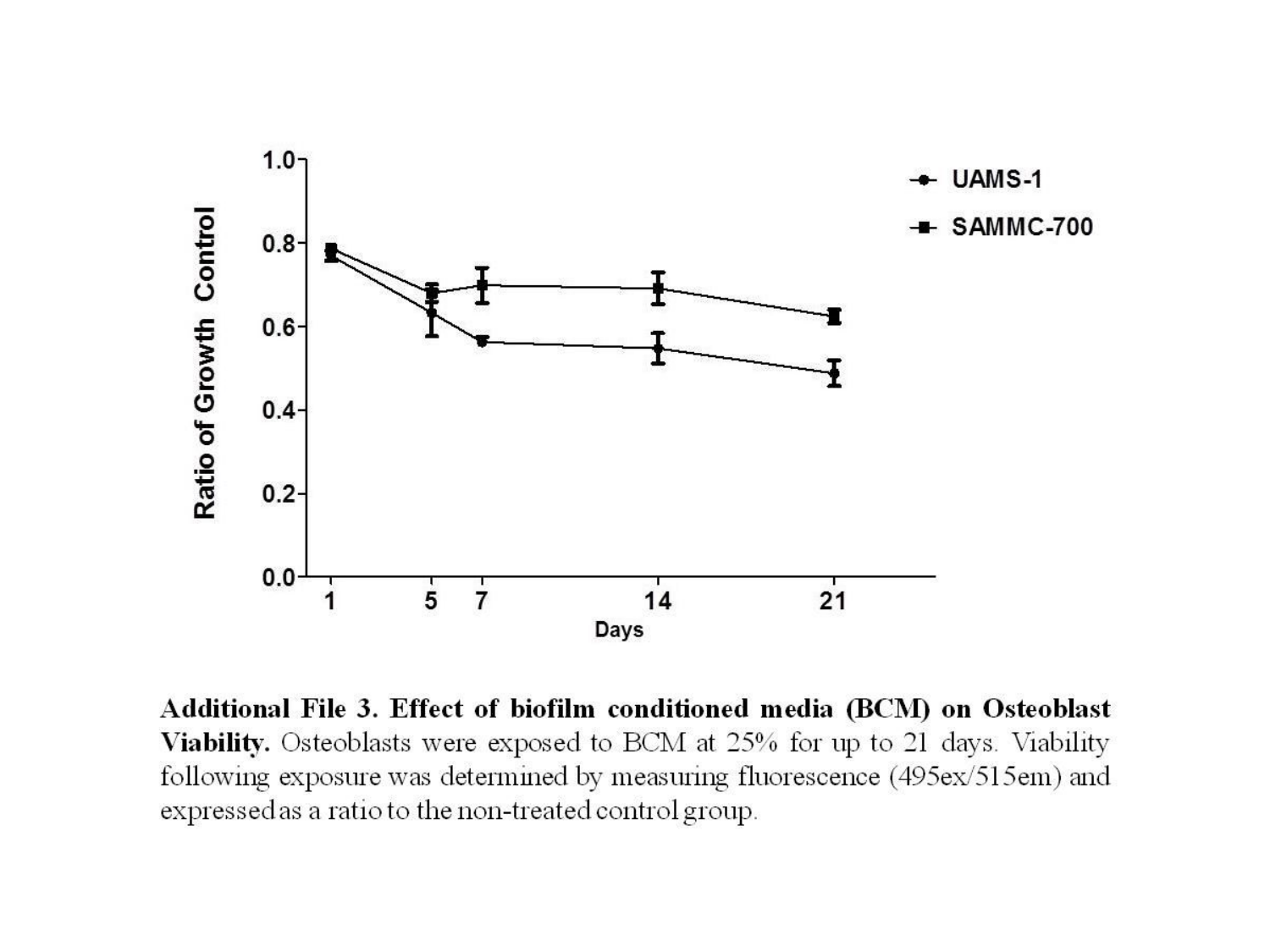

Supplement: Additional file 3 — Effect of biofilm conditioned media (BMC) on Osteoblast Viability. Osteoblast were exposed to BMC at 25% for up to 21 days. Viability following exposure was determined by measuring fluorescence (495ex/515em) and expressed as a ratio to the non-treated control group. [file 1471-2474-14-187-S3.pptx]
